# The *GALNS* p.P77R variant is a probable Gujarati-Indian founder mutation causing Mucopolysaccharidosis IVA syndrome

**DOI:** 10.1186/s12864-022-08693-4

**Published:** 2022-06-21

**Authors:** Harsh Sheth, Premal Naik, Maulin Shah, Riddhi Bhavsar, Aadhira Nair, Frenny Sheth, Jayesh Sheth

**Affiliations:** 1 FRIGE’s Institute of Human Genetics, FRIGE House, Jodhpur Village Road, Satellite, Ahmedabad, Gujarat 380015 India; 2 Rainbow Super Speciality Hospital and Children’s Orthopedic Centre, Ahmedabad, India; 3 Orthokids Clinic, Ahmedabad, India

**Keywords:** Mucopolysaccharidosis IVA (MPS IVA), Morquio A syndrome, *GALNS*, Founder variant, p.P77R

## Abstract

**Background:**

Mucopolysaccharidosis IVA (Morquio syndrome A, MPS IVA) is an autosomal recessive lysosomal storage disorder caused due to biallelic variants in the N-acetylgalactoseamine-6-sulfate sulfatase (*GALNS*) gene. The mutation spectrum in this condition is determined amongst sub-populations belonging to the north, south and east India geography, however, sub-populations of west Indian origin, especially Gujarati-Indians, are yet to be studied. We aimed to analyse the variants present in the *GLANS* gene amongst the population of Gujarat by sequencing all exons and exon–intron boundaries of the *GALNS* gene in patients from 23 unrelated families.

**Results:**

We report 11 variants that include eight missense variants: (p.L36R, p.D39G, p.P77R, p.C79R, pP125L, p.P151L, p.G255A and p.L350P), one splice site variant: (c.121-7C > G), one small insertion: (c.1241_1242insA, p.I416HfsTer2) and one small deletion: (c.839_841delACA). Of these, three missense variants (p.D39G, p.G255A and p.L350P), one splice site and the two indels mentioned above are novel. Interestingly, we observed a higher than anticipated prevalence of p.P77R variant in our cohort (*n* = 14/25, 56%). Haplotype analysis in cases with p.P77R variant and 63 ethnicity matched healthy population controls suggested a 4 SNP haplotype block present in cases compared to controls (likelihood ratio test *p*-value = 1.16 × 10^–13^), thereby suggesting p.P77R variant as a founder variant in the Gujarati-Indian population. Furthermore, age of mutation analysis suggested the variant to have arisen approximately 450 years ago in the population.

**Conclusion:**

p.P77R variant in the *GLANS* gene is likely to be a founder variant in MPS IVA patients of Gujarati-Indian ancestry and appeared approximately 450 years ago in the population. To our knowledge, this is the first variant to be posited as a founder variant in the *GLANS* gene in patients with MPS IVA syndrome.

**Supplementary Information:**

The online version contains supplementary material available at 10.1186/s12864-022-08693-4.

## Background

Mucopolysaccharidosis IV A (MPS IVA, Morquio-A syndrome, OMIM#253,000) is an autosomal recessive disorder which is caused by the deficiency in enzyme activity of N-acetylgalactosamine-6-sulfate sulfatase (GALNS) due to biallelic variants in the *GALNS* gene (OMIM#612,222) [1, 2]. GALSN enzyme plays an essential role in the degradation of glycosaminoglycans (GAGs), keratan sulfate (KS) and chondroitin-6-sulfate (C6S) [3]. Therefore, deficiency in the GALNS enzyme activity leads to the accumulation of these substrates in the lysosomes, particularly in cartilage and cornea, leading to a wide gamut of clinical manifestations such as shorth trunk, short stature, genu valgum, pectus carinatum, kyphoscoliosis, joint laxity, dysmorphic face, hepatomegaly and abnormal gait [4].

MPS IVA is a rare lysosomal storage disorder, with an estimated incidence ranging from one in 76,000 in Northern Ireland to one in 640,000 births in Western Australia [4–6]. Although the study in the Asian population is scarce, the available data estimates the birth prevalence of MPS IVA to be 1 in 500,000 live births in Japan [7], 1 in 304,000 in Taiwan [8], and 1 in 701,000 live births in Malaysia [9].

The *GALNS* gene is situated on chromosome 16q24.3 and comprises of 14 exons spanning over 50 kb of genomic length and codes for a 522 amino acid GALNS protein with a signal peptide of 26 amino acids [10]. To date, a total of 333 variants in the *GALNS* gene have been reported which comprises of 248 missense/ nonsense variants, 32 small deletions, 5 small insertions, 2 small indels, 32 splice site variants and 3 complex rearrangements in the HGMD database [http://www.hgmd.org, accessed on 21^st^ October 2021]. Of these, the 10 most commonly reported variants in MPS IVA patients from across the globe are: c.120 + 1G > A, c.337A > T, c.757C > T, c.860C > T, c.871G > A, c.901G > T, c.935C > G, c.953 T > G, c.1156C > T and c.1171A > G [11]. Some of these variants are also commonly observed in particular sub-populations such as c.120 + 1G > A in 91% of Tunisian patients, c.1171A > G and c.337A > T in 64% and 52% of the Irish patients respectively, c.757C > T in 89% of Pakistani patients and c.953 T > G in 58% of Chinese patients [11].

Interestingly for the Indian population, a large study of 68 unrelated MPS IVA patients from mostly northern and southern geographical regions identified 22 novel variants [12]. Of these, c.860C > T (8.82%), c.647 T > C (7.35%), c.95A > C (6.61%) and c.871G > T (5.88%) were the most frequent variant in the observed population compared to other populations [12]. However, considering the diverse genetic architecture of the sub-populations residing in the Indian sub-continent, the study is likely to miss variants predominant in patients from western and eastern parts of India.

Here, we present the mutation spectrum of the *GALNS* gene in 23 patients from unrelated families from the western part of India, predominantly Gujarat. Furthermore, we present evidence for p.P77R variant as a founder variant in the Gujarati-Indian sub-population through haplotype analysis and estimate its age of emergence in the population.

## Results

### Clinical and GALNS enzyme activity spectrum

A total of 23 patients affected with MPS IVA were included in the present study. Out of the 23 families, seven had consanguineous marriages and the remaining 16 families practised endogamy. The age at diagnosis ranged from 11 months to 21 years with a mean age of 4.32 years. Detailed clinical and anthropometric information is presented in Table 1. Common clinical features included: short stature, short fingers, short neck-trunk, and frontal bossing. Kyphosis was seen in 39% of the patients (*n* = 9/23) and platyspondyly in 21% of the patients (*n* = 5/23). Of note, other prominent features of MPS IVA such as corneal clouding and knock knee were seen in 1/23 (5%) and 7/23 (30%) patients, respectively. On the radiological assessment of the patients, the key findings were anterior beaking of the vertebral bodies, acetabular dysplasia, platyspondyly, and multiple dysplastic epiphyses. Due to the absence of growth parameters for these patients, we could not classify the disease severity.

**Table 1:** Clinical and anthropometric details of MPS IVA patients in the present study

**Patient ID**	**Age (in years)**	**Sex**	**Religion (Region)**	**Consanguinity**	**Phenotype**							**Additional features**	**GALNS enzyme activity (nmol/hr/mg protein) [NR: 14–32 nmol/hr/mg protein]**
					**Short stature**	**Pectus carinatum**	**Kyphosis**	**Scoliosis**	**Genu valgum**	**Platyspondyly**	**Anterior beaking of bone**		
P1	3	Female	Hindu (Gujarat)	No	-	-	✔	-	-	-	✔	Short fingers, strabismus, hepatomegaly	6.0
P2	1.9	Male	Hindu (Gujarat)	No	-	✔	✔	-	-	-	✔	Lumbar lordosis, upper femoral epiphysis, pleural effusion	3.7
P3	0.9	Female	Muslim (Gujarat)	Yes	-	-	✔	✔	-	-	-	Gibbus	0.75
P4	1.5	Male	Muslim (Gujarat)	Yes	-	-	-	-	-	-	✔	Pointed appearance of bones of metacarpals of both hands, bilateral deformity of lower ends of radius and ulna, distal ends of proximal phalanges of fingers are pointed	11.1
P5	8	Female	Hindu (Gujarat)	No	✔	-	✔	-	✔	-	✔	Skeletal deformity, extended neck, delayed motor development, frontal bossing, corneal clouding, short neck and trunk, widening of wrist and elbow, mild hypotonia, distal ends of proximal phalanges of fingers are pointed, shortening of metacarpal bones	4.8
P6	1	Female	Hindu (Gujarat)	No	-	-	✔	✔	-	✔	✔	Large skull, frontal bossing, wide ribs, bullet shaped phalanges, wide ribs	4.15
P7	2	Male	Hindu (Gujarat)	No	✔	-	✔	-	-	-	-	Hydrocephalus	4.3
P8	6	Male	Darbar (Gujarat)	Yes	✔	✔	-	✔	✔	-	-	Rickets	5.6
P9	2	Male	Hindu (Gujarat)	No	-	-	✔	-	-	✔	-	Bullet-shaped metacarpals, bilateral hip dysplasia	7.7
P10	1	Male	Hindu (Gujarat)	No	-	-	✔	-	-	✔	-	Abnormal bilateral capital femoral epiphysis	3.0
P11	6	Male	Hindu (Gujarat)	No	✔	-	-	-	-	-	-	Short neck and short fingers	1.1
P12	1.7	Female	Hindu (Gujarat)	No	-	-	-	-	-	-	-	Coarse face, skeletal abnormality, gait disturbance	0.2
P13	2.5	Male	Hindu (Gujarat)	No	-	-	-	-	✔	✔	✔	Bullet-shaped phalanges, shortening of metacarpal bones, subluxation of distal radio-ulna joint, distorted/ sclerotic shallow acetabulum	0.05
P14	3.7	Female	Hindu (Gujarat)	No	✔	-	-	-	✔	-	✔	Acetabulum dysplasia, epiphyseal dysplasia, prominent metaphysis mainly at knee and wrist, ulnar deviation at wrist, short palm and fingers, bullet shaped metacarpals	0.37
P15	4	Female	Hindu (Gujarat)	No	✔	-	-	-	-	-	✔	Large skull, short and wide metacarpals with pointed base, Madelung deformity in wrist, wide gait, shoulder contractures	0.08
P16	12	Female	Hindu (Gujarat)	No	✔	✔	-	-	-	✔	-	Bilateral lower and upper limb weakness, deformity of upper limbs, unable to walk, spinal canal narrowing, deep acetabulum, corneal opacity	3.4
P17	21	Male	Hindu (Gujarat)	No	-	-	-	-	-	-	-	Skeletal dysplasia	0.03
P18	5	Male	Javia (Gujarat)	No	✔	-	-	-	✔	-	-	Ankle deformity, bilateral Plano valgus feet, spondylo epiphyseal dysplasia	0.25
P19	3.5	Male	Muslim (Gujarat)	Yes	✔	-	-	-	-	-	-	Dysostosis multiplex, Coarse face	0.04
P20	4	Female	Muslim (Gujarat)	Yes	✔	-	-	-	✔	-	-	Lumbar lordosis, wrist widening, bilateral flattened sclerotic femoral head	0.8
P21	1.6	Male	Hindu (Gujarat)	No	-	-	✔	✔	-	-	✔	Short metacarpals with pointed proximal ends, mild narrowing of foramen magnum with cord compression and focal significant cord oedema at C1-C2 level	0.02
P22	6	Female	Muslim (Gujarat)	Yes	-	✔	-	-	✔	-	-	Coarse face, increased wrist angle	0.025
P23	2	Female	Muslim (Gujarat)	Yes	-	-	-	-	-	-	-	Skeletal dysplasia	0.33

*NR* Normal range

Elevated levels of urinary GAG were observed in all the patients with excess keratan sulfate (KS) and chondroitin sulfate (CS). The enzyme activity of N-acetylgalactosamine-6-sulfate sulfatase in all the patients was in the range of 0.02–11.1 nmol/hour/mg of protein and the mean enzyme activity was 2.30 nmol/hour/mg of protein which was less than 10% of the mean normal enzyme activity (Table 1).

### *GALNS* gene mutation spectrum

**Fig. 1:**
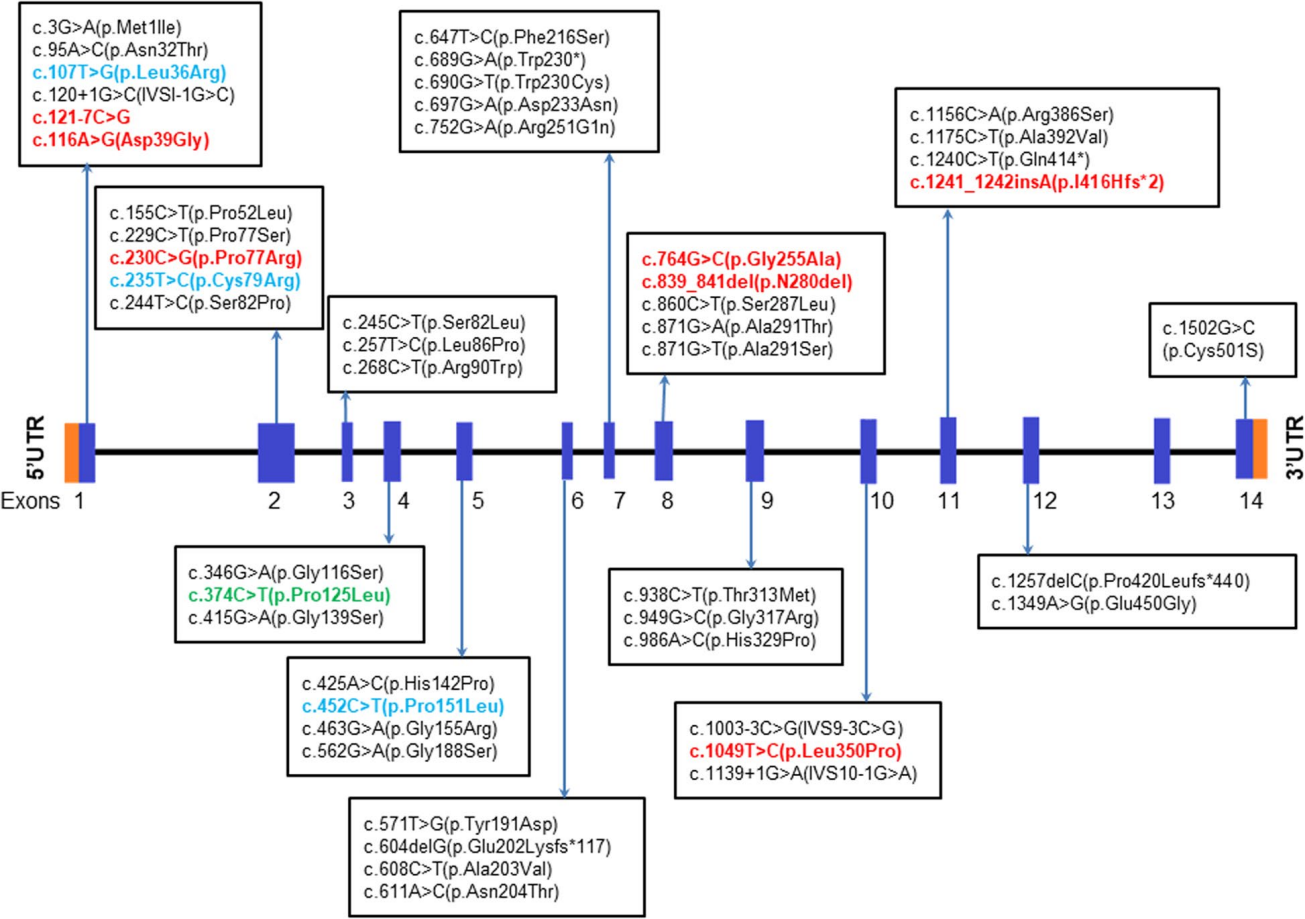
Schematic representation of the variants identified in the *GALNS* gene in patients with MPS IVA from India to date. Variants highlighted in red are observed in the present study whereas variants highlighted in blue are reported by Bidchol et al. 2014 [12] and the present study. Variants highlighted in green has reported in the Chinese population and the present study

The 23 patients harboured 11 different variants in the *GALNS* gene of which, 8 were missense variants (84%): c.107 T > G (p.L36R), c.116A > G (p.D39G), c.230C > G (p.P77R), c.235 T > C (p.C79R), c.374C > T (pP125L), c.452C > T (p.P151L), c.764G > C (p.G255A) and c.1049 T > C (p.L350P), 1 was a splice site variant (4%): c.121-7C > G, one small insertion (4%): c.1241_1242insA (p.I416HfsTer2) and one small deletion (4%): c.839_841delACA (Table 2; Fig. 1). Of these, 3 missense, one splice site and two indel variants were novel (p.D39G, p.G255A, p.L350P, c.121-7C > G, c.839_841delACA and c.1241_1242insA/p.I416HfsTer2) to our population (Table 2; Fig. 1). All 23 patients were observed to have a variant in the homozygous state except in two patients where the variants were likely to be compound heterozygous (c.230C > G (p.P77R)/ c.764G > C (p.G255A) and c.839_841delACA (p.N280del)/ c.374C > T (p.P125L); Table 2; Fig. 1). All known and novel variants were classed as pathogenic/ likely pathogenic according to the ACMG-AMP classification system [13].

**Table 2:** *GALNS* gene mutation identified in Gujarati-Indian MPS IVA patients using Sanger sequencing

**Patient ID**	**cDNA change**^**a**^	**Amino acid change**^**b**^	**Location**^**c**^	**Mutation type**	**Allele frequency (gnomAD**^**d**^**)**	**Ethnicity**	**Reference**	**PolyPhen-2 analysis**	**DANN score**	**ACMG-AMP classification**
P20	c.121-7C > G	-	Intron 1	Splice site	NA	Indian	Present study	-	-	Uncertain significance/ likely pathogenic
P4, P19	c.107 T > G	p.L36R	Exon 1	Missense	0.00003	Southeast Asian- multi-ethnic, European	Morrone et al. 2014 [11], Caciotti et al. 2015 [14]	1 Probably damaging	0.9889	Pathogenic
P7	c.116A > G	p.D39G	Exon 1	Missense	NA	Indian	Present study	0.997 Probably damaging	0.9844	Likely pathogenic
P1-P3, P5, P9, P10, P11^e^, P12, P14, P17, P18, P21-P23	c.230C > G	p.P77R	Exon 2	Missense	NA	Indian, Turkish	Tomatsu et al. 1995 [15], Present study	1 Probably damaging	0.9984	Pathogenic
P16	c.235 T > C	p.C79R	Exon 2	Missense	0.00000807	Indian, Malaysian	Bidchol et al. 2014 [12], Leong et al. 2019 [9]	1 Probably damaging	0.9974	Pathogenic
P15^e^	c.374C > T	p.P125L	Exon 4	Missense	0.00000399	Chinese	Zhao et al. 2011 [16]	1 Probably damaging	0.9247	Pathogenic
P8	c.452C > T	p.P151L	Exon 5	Missense	0.0000279	Indian	Bidchol et al. 2014 [12]	1 Probably damaging	0.9986	Pathogenic
P11^e^	c.764G > C	p.G255A	Exon 8	Missense	NA	Indian	Present study	1 Probably damaging	0.9979	Likely pathogenic
P15^e^	c.839_841delACA	p.N280del	Exon 8	Deletion	NA	Indian	Present study	-	-	Likely pathogenic
P13	c.1049 T > C	p.L350P	Exon 10	Missense	NA	Indian	Present study	1 Probably damaging	0.9989	Likely pathogenic
P6	c.1241_1242insA	p.I416Hfs^e^2	Exon 11	Frame shift	NA	Indian	Present study	-	-	Pathogenic

*NA* Not available, *DANN* Deep learning approach to annotating variants

^a^cDNA numbering is based on RefSeq transcript NM_000512.5

^b^Amino acid change is based on NP_00503.1

^c^Genomic position is based on hg19/GCRh37 genome build

^d^gnomAD allele frequency is based on version 2.1

^e^Patient was heterozygous for the said variant

Interestingly, whilst the c.229C > T (p.P77S) variant had previously been reported in MPS IVA patients from India [12], the proportion of patients with another variant at the same amino acid residue c.230C > G (p.P77R) was significantly high in our cohort (60%; Table 2). Since our cohort was primarily derived from the same geo-ethnicity, a substantially higher than expected prevalence of the p.P77R variant in the cohort suggested it to be a potential founder variant.

### *GALNS* p.P77R haplotype and age of variant estimation

**Fig. 2:**
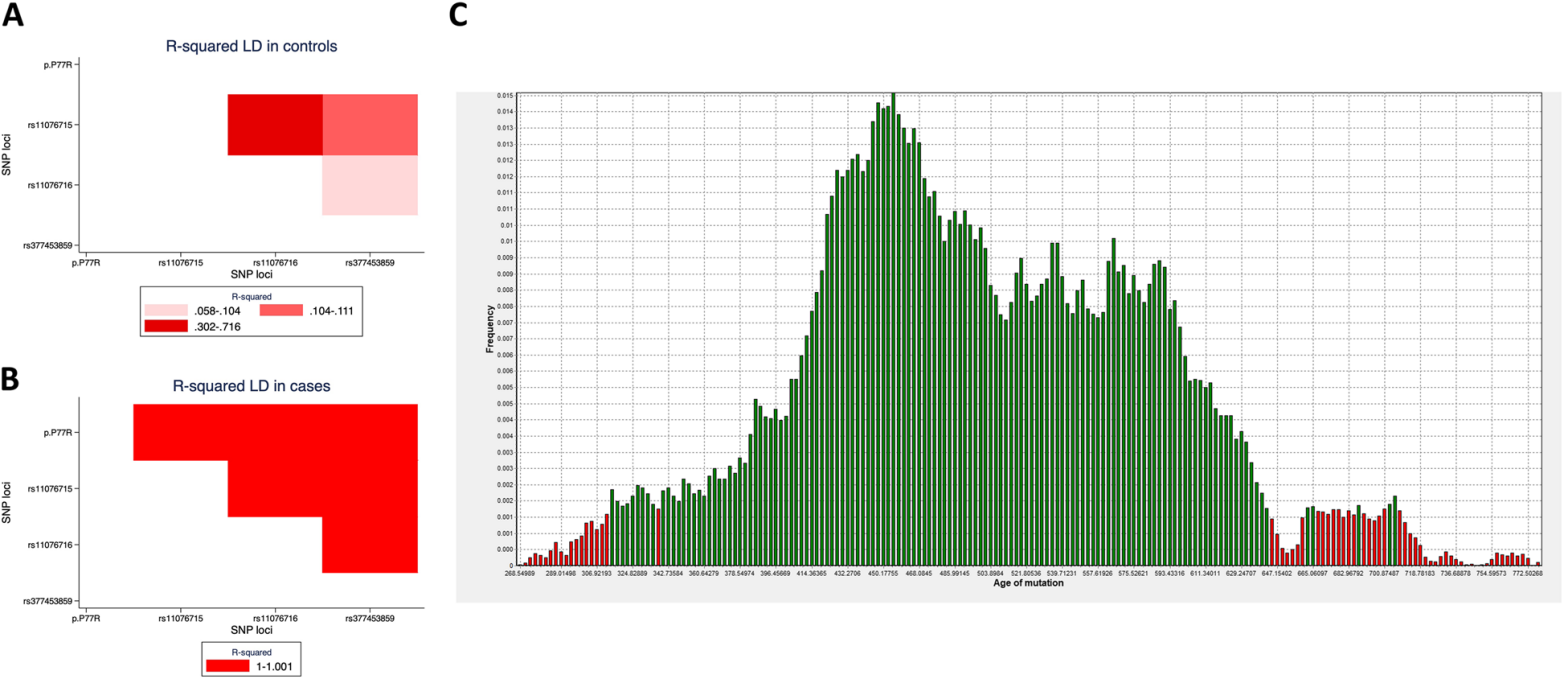
Linkage disequilibrium, haplotype and age of variant analysis for p.P77R variant in the *GALNS* gene in Gujarati-Indian population. Linkage disequilibrium plot in **A** 63 controls **B** 13 cases with p.P77R variant in the *GALNS* gene. Red colour intensity represents strength of linkage disequilibrium (measured in *R*^2^) between 2 SNPs. **C** Predicted age of variant plot generated by the DMLE software. Green indicates values within 95% confidence intervals

A total of 31 SNPs were found between exons and exon–intron boundaries of the *GALNS* gene using the smMIP based sequencing approach within 13 cases and 63 controls. The haplotype associated with the p.P77R variant were formed by the SNP markers rs11076715, rs11076716 and rs377453859 with a total genomic length of 28.3 kb (Fig. 2A and B). We observed a significant enrichment for this haplotype in cases compared to controls (likelihood ratio test *p*-value = 1.16 × 10^–13^), suggesting the p.P77R to be a founder variant in the MPS IVA patients of Gujarati-Indian ethnicity. Analysis performed by the DMLE + 2.3 software estimated that the age of the *GALNS* p.P77R variant might be approximately 450 years (95% CI: 306–647 years) (Fig. 2C) in the Gujarati-Indian population.

## Discussion

Mutational spectrum analysis for a given disease in diverse populations have provided critical evidence for understanding disease pathophysiology, gene domains that are intolerant to variation and development of therapeutic strategies. Indeed, communities that are geographically isolated or practice consanguinity/ endogamy are likely to have founder variants. Identification of these variants are critical for developing and deploying targeted molecular diagnostics which could be used for initial screening, prenatal testing, reducing costs and turnaround time of tests [17]. Founder variants exhibit linkage disequilibrium with nearby genetic markers. The size of the linkage disequilibrium interval is inversely correlated with the time since the variant emerged in a given population and studying the prevalence of a given founder variant amongst different sub-populations could provide evidence for their shared ancestry [18].

The current study is the first to report the mutation spectrum within the *GALNS* gene in patients diagnosed with MPS IVA disease from the western part of India, especially Gujarat. Whilst ours is not the first study from India [12], the difference in the mutational spectrum between the two studies suggests either lack of or absence of patient cohort from the western part of India in the previous study. Furthermore, subtle differences in the phenotypic manifestation such as the mean age of the patient cohort (4.32 years in the current study versus 6.7 years in Bidchol et al.) could either be due to the method of recording the age (i.e. age at diagnosis in the current study versus age of presentation in Bidchol et al.) or manifestation of clinical phenotypes (severe or attenuated phenotype) which is age dependent. Another important aspect for consideration is that the majority of the patients on the current study were clinically assessed over 10 years ago and were not amenable to follow-up. Therefore, it is plausible that a detailed phenotype data capture may not have occurred in the current study and could lead to the differences in the reported phenotypes between both studies.

However, there are several grounds of congruence between the two studies, such as, similar prevalence of missense variants between the two studies (~ 80%). Indeed, the mutation data for the *GALNS* gene in the HGMD database also shows missense variants to be the most common. Out of the eight missense mutations identified in our study, five have been previously reported in MPS IVA patients from different populations (p.L36R, p.P77R, p.C79R, p.P125L and p.P151L). The mutation p.C79R was first reported in an Indian MPS IVA patient by Bidchol et al. followed by Leong et al. who reported the same mutation in an Indian patient from Malaysia [9, 12]. Likewise, p.P125L and p.P151L have been previously reported in Chinese and Indian populations [12, 16]. Structural studies have shown the amino acid Proline at the 125^th^ position to be located in the N-cap domain of the alpha-helix. Also, the amino acid Proline at the 151^st^ position is a conserved amino acid. Hence, any substitution at these positions is likely to result in modification of the packing and disturbing the tertiary structure of the GALNS protein [19]. The two most common variants identified in the present study cohort were p.L36R and p.P77R. The p.L36R variant reported by Bidchol et al. is predicted in silico to affected secondary structures and hydrophobic core of the GALNS protein [12], whereas another missense change at the same amino acid residue p.L36P is associated with an attenuated phenotype as reported by Tomatsu et al. [20]. In congruence with this, the patients in our cohort with this variant presented with relatively milder phenotype. The variant p.P77R was first reported by Tomatsu et al. in a MPS IVA patient of Indian ethnicity [15]. This mutation has been reported in patients with severe phenotype [21]. Also, the amino acid Proline at position 77 has a key role in preventing the internalization in the hydrophobic patch of the GALNS protein. The positively charged side chain of arginine instead of proline is expected to have a destabilizing effect in this process [19]. Interestingly, none of the ten most common mutations in the *GALNS* gene mentioned in the earlier reports were found in our study. This observation is similar to that made by Bidchol et al. in their cohort of Indian patients [12]. This gives strong evidence about the molecular heterogeneity in India as well as the presence of a distinct mutation spectrum for the Indian sub-populations.

Haplotype analyses have previously been conducted in lysosomal storage disorders to identify founder variants, for example, *GBA* p.N370S in Ashkenazi Jewish population [22] and *GBA* p.G85E in Korean population [23]. Indeed, our group has previously identified a common variant p.E462V in the *HEXA* gene in Tay Sach disease patients of Gujarati Indian ethnicity [24]. However, to our knowledge, no founder variant has been identified to date for MPS IVA patients throughout the world. To demonstrate that the variant p.P77R emerged due to a founder effect, a total of 30 SNPs were analysed of which 3 SNPs formed a strong linkage disequilibrium with the variant, which was observed across all 13 unrelated families in the current study. This variant may have emerged within the population itself or may have been brought through immigration and subsequent population bottleneck. However, due to the lack of mutational data availability from sub-populations residing in geographical units around Gujarat, it is difficult to assess the emergence of this variant and estimate number of carriers for this variant outside Gujarat. The Indian population is highly heterogeneous with each sub-population likely practicing endogamy/ consanguinity, hence, an accurate method for determination of the ethnicity of the studied individuals would be with the use of high-density SNP arrays, an analysis which is beyond the scope of the present study.

Technological advances coupled with population specific genetic architecture details provides a valuable tool for an efficient diagnosis and screening strategies for rare diseases like MPS IVA. In our study, we used urinary GAG as a screening test which is a relatively inexpensive approach for detection of MPS IVA patients. This screening test coupled with typical phenotypic features and founder mutation testing is likely to help towards rapid and low-cost diagnosis of MPS IVA patients in Gujarat. With the Gujarati-Indians forming large diasporas in several western nations including the USA and UK, the impact of the current study on MPS IVA diagnostics is likely to be profound beyond India.

We hereby provide the first report showcasing the mutation spectrum in the *GALNS* gene amongst MPS IVA patients of the Gujarati-Indian origin. Furthermore, we provide evidence of a founder effect for the p.P77R variant in the Gujarati-Indian population with an estimated age of the variant to be approximately 450 years, which can be used as first-line marker for rapid genetic diagnosis in this population.

## Materials and methods

### Patient recruitment

The present study comprises of 23 unrelated families with at least one member with clinical suspicion of MPS IVA (*N* = 23). These patients were recruited on the study after obtaining written informed consent from their parents/ guardians. The patients were clinically suspected of MPS IVA and their diagnosis was confirmed by the presence of keratan sulfate in urinary GAG one dimensional electrophoresis study followed by beta-galactosidase sulfate assay. Clinical history, family history and consanguinity details were recorded in pre-designed clinical *pro forma.* Sixty-three unrelated individuals from the same ethno-geographic group were recruited as controls. The study protocol was approved by the Institutional Ethics Committee of the Foundation for Research in Genetics and Endocrinology, Ahmedabad (Registration no: E/13237) as per the Helsinki Declaration. 5 ml of peripheral whole blood was collected from cases and controls for GALNS enzyme and molecular diagnostic assays. Furthermore, 10-15 ml of urine sample was collected from cases for urinary GAG testing.

### Urinary GAG testing and GALNS enzyme assay

Urinary GAG quantitative study was performed using dimethylmethylene blue dye based spectroscopic method [25]. The concentration of urine creatinine of individual patients was measured using Liquixx Creatinine Kit (Erba Mannheim, Germany) as per the manufacturer’s instructions. The pre-treatment of urine prior to the qualitative analysis of GAGs by electrophoresis was performed according to the protocol by Hopwood and Harrison 1982 [26]. Lysosomal enzyme β-galactose-6-sulfate-sulfatase activity was carried out from leukocytes using fluorogenic synthetic substrate 4-methylumbelliferyl-β-galactose-6-sulfate-triethyl ammonium as described by Van Diggelen et al., 1990 [27]. The fluorescence of free 4-methylumbelliferone (4 MU) was measured by LS55 spectrofluorometer (Perkin Elmer, USA) to determine the β-galactose-6-sulfate-sulfatase activity. Protein concentration was determined by the Lowry method. The enzyme activity was expressed as the amount of substrate in nmol cleaved per hour per mg of protein in the cell lysates. Normal range for the GALNS enzyme activity was 14–32 nmol/hr/mg of protein.

### *GALNS* gene mutation identification by Sanger sequencing

For all cases and controls, high molecular weight genomic DNA was isolated from peripheral blood by the salting-out method [28]. Exon and exon–intron boundaries of the *GALNS* gene were amplified by PCR in 14 fragments using 14 primer pairs (Supplementary Table 1). A 10 μl reaction mixture for the DNA amplification of each fragment was made up of 100 ng genomic DNA, 1 mM dNTPs, and 10X Cetus buffer. 30 cycles of amplification were performed, each consisting of denaturation at 94℃ for 1 min, annealing at 60–65℃ suitable for each exon for 45 s, and extension at 72℃ for 45 s in a thermal cycler. The final extension time was at 72℃ for 10 min. PCR products along with a 100 base-pair DNA ladder were then subjected to electrophoresis in 2% agarose gel for validation of amplification and the amplified products were purified using Exo-SAP-IT™ (USB Corporation, OH, USA). The purified products were sequenced using BigDye Terminator v3.1 and capillary electrophoresis was performed using an automated sequencer ABI-3500 (Applied Biosystems, CA, USA) for mutation analysis of *GALNS*. Bi-directional sequencing data was analysed by comparing the sequence read with the reference sequence of the *GALNS* gene (RefSeq cDNA NM_000512.5). Identified variants were annotated with data from 1000genomes, gnomAD, dbSNP and the Human Gene Mutation Databases. In silico assessment of variant pathogenicity was carried out using SIFT (Sorting Intolerant From Tolerant) (http://sift.jcvi.org), Polyphen2 (Polymorphism Phenotyping v2) (http://genetics.bwh.harvard.edu/pph2/), MutPred (http://mutpred.mutdb.org/), PROVEAN (http://provean.jcbi.org) and MutationTaster (http://www.mutationtaster.org/). Finally, variants were annotated for pathogenicity using the ACMG-AMP classification system for single nucleotide variants [13].

### SNP genotyping in the *GANLS* gene for haplotype analysis of p.P77R variant

A total of 12 out of 14 cases harbouring p.P77R variant and 63 controls were genotyped for common single nucleotide polymorphisms (SNPs) in the *GALNS* gene for haplotype analysis using targeted capture by single molecule molecular inversion probe (smMIP) based sequencing assay. Patients P14 and P21 were not included in the analysis due to the lack of adequate amount of genomic DNA required for the assay. Additionally, an MPS IVA patient of Gujarati-Indian ethnicity was included in the haplotype analysis as the patient had previously been identified with a heterozygous p.P77R variant, although, clinical phenotype data was unavailable. Briefly, smMIPs targeting exons and exon–intron boundaries of the *GANLS* gene were designed using the MIPgen tool [29]. smMIP probes were designed against the GCRh37/hg19 human reference genome build with following set of parameters: a target capture size of 110 bp, a combined length of 40 bp for the targeting arms, 5 bp unique molecular barcode sequence and no common single nucleotide polymorphisms (SNPs; dbSNP151 database) in the smMIP extension or ligation arm. A total of 40 smMIPs were designed (Supplementary Table 2). The smMIP capture was performed in accordance with the protocol described, with minor modifications [30]. In short, the regions of interest were captured in a reaction containing a molecular ratio between genomic DNA and smMIP of 1:1000. The smMIP capturing conditions were 10 min at 95 °C for denaturation, followed by an incubation period of 16–18 h during which hybridization of the phosphorylated smMIPs to the single stranded target DNA occurs together with gap-fill and probe circularization via ligation. All non-circular targets were digested by exonuclease treatment and the circular targets were amplified with primers containing sample specific barcoded reverse primers with following PCR conditions: 30 s at 98 °C followed by 20 cycles of 10 s at 98 °C, 30 s at 60 °C and 30 s at 72 °C and 2 min at 72 °C. Primers used for amplification contained adapters compatible with Illumina sequencing [31]. After PCR, libraries were pooled each from 76 barcoded individual libraries. The pooled libraries were purified using Agencourt AMPure XP beads according to the manufacturer’s protocol (Beckman Coulter, USA) and the final library was diluted to a concentration of 9 pM and subsequently sequenced on a MiSeq platform (Illumina, USA) according to the manufacturer’s protocol (300 cycles; V3 kit) resulting in 2 × 156 bp paired end reads.

Data was analysed using an in-hose smMIP pipeline which involved: trimming of 5 bp unique molecular barcode (UMB) from fastq files and stored for later use, read alignment against the GCRh37/hg19 human reference genome using BWA mem (v0.7.12) [32] with output presented as a sample specific BAM file amalgamated with UMB data, base quality score recalibration using GATK v4.1.12 and single nucleotide variants and indel calling using GATK’s HaplotypeCaller v4.1.12 to create a VCF file. Data from VCF file was annotated with 1000 genomes and gnomAD databases to identify common SNPs (> 5% minor allele frequency) in the general population and the filtered SNPs were then transposed to a multi-sample excel sheet (Supplementary File 1).

### Haplotype reconstruction and age of p.P77R variant

All analyses were carried out using GCRh37/hg19 chromosomal positions. SNPs along the coding regions of the *GALNS* gene were sued to obtain the haplotype that flanks the p.P77R variant in 13 cases and 63 controls. SNPs were assessed for Hardy–Weinberg equilibrium within the control cohort and any SNP not equilibrium was not assessed in the downstream analyses. Pairwise linkage disequilibrium using -pwld- command was used to estimate SNPs in linkage disequilibrium (*R*^2^ score). Association of the linkage disequilibrium block between cases and controls was assessed using the likelihood ratio test and -hapipf- command. A two-sided *p*-value of < 0.05 was considered statistically significant. All haplotype analysis was carried out using Stata v12. A 4 SNP haplotype block was selected for variant dating. The DMLE + 2.3 software [33] was used to estimate the age of p.P77R variant. The algorithm uses an intra-allelic coalescent model to assess the linkage disequilibrium across the marker set coupled to marker locations, population growth rate (0.01 for India), proportion of population samples (0.00248 for Gujarat) and a proportion of disease bearing chromosomes. Full input details are available in the Supplementary File 2.

## Supplementary Information

**Additional file 1: Supplementary Table 1.** List of primer pairs used to amplify exon/ exon intron boundaries of the *GALNS* gene for Sanger sequencing.

**Additional file 2: Supplementary Table 2.** List of single molecule molecular inversion probes for target capture of exons and exon-intron boundaries of the *GALNS* gene.

**Additional file 3: Supplementary File 1.** Genotyped SNP output data from 13 MPS IVA patients and 63 controls used for haplotype analysis.

**Additional file 4: Supplementary File 2.** Input file for DMLE software for age of variant analysis.

## Data Availability

Raw data used for haplotype analysis and mutational age estimation is provided as supplementary files. Furthermore, raw sequence files generated from smMIP based targeted sequencing can be accessed from the European Nucleotide Archive (Project Accession: PRJEB51874) using the link https://www.ebi.ac.uk/ena/browser/search.
