# Supplementary material for: The GALNS p.P77R variant is a probable Gujarati-Indian founder mutation causing Mucopolysaccharidosis IVA syndrome
Source: BMC Genomics. 2022 Jun 21;23:458. doi: 10.1186/s12864-022-08693-4 (PMC9210747; doi:10.1186/s12864-022-08693-4)
Supplement: Supplementary file 1 — Additional file 1: Supplementary Table 1. List of primer pairs used to amplify exon/ exon intron boundaries of the GALNS gene for Sanger sequencing. [file 12864_2022_8693_MOESM1_ESM.docx]

**Supplementary Table 1: List of primer pairs used to amplify exon/ exon intron boundaries of the *GALNS* gene for Sanger sequencing.**

| **EXON** | **Forward Primer** | **Reverse Primer** |
| --- | --- | --- |
| 1 | ACTGGTCACGAGGCAGTC | GCGTGTGGTGATCGGTGA |
| 2 | GTGCTGGCTTCCCACGGTC | CAGAGTCAGGGCTGGAAGGA |
| 3 | CGTCTGTCACGCGTCTGT | ACCAGCGGTACCCCACCT |
| 4 | GAGGGGCCAGTGTCCTGT | GCTGGAGACACCTGAACACA |
| 5 | TGAGGCCTTGGTCTTTTGTC | ACTTGAGCCCACCAGTGCTA |
| 6 | CCAGCCCTAGCCTTCTTTTA | AGGTTGATGCATTCCTGTCC |
| 7 | GGTTCAGGGACCTCATCACC | TTGCTCTGGCCTTTCCAT |
| 8 | TGAACTGAGGCCATTCCTCT | GTCACTCCTGCTTCCAGGTT |
| 9 | GGCCCTTTGTCCCTATGAC | GAGGGTGGTGAGGCTGAG |
| 10 | TGGTTTGAGGCTCCTCTGTC | CCCCAGGTCCTCTCGTCT |
| 11 | GTATCAACCAAGACCTCACG | TGGAGTTCCTGCCTGTCTCA |
| 12 | CTAGGCACAGGCAGACGAGA | CAAGCACGTGTGGGTATGAA |
| 13 | GTGGTTCTCAGCCCGTTAGA | TGTGCTCTGAGGCACGAG |
| 14 | CAGAACAGCAACAACAAAATGC | AACTGTATCCCCAGCCACCT |
