# Supplementary material for: The GALNS p.P77R variant is a probable Gujarati-Indian founder mutation causing Mucopolysaccharidosis IVA syndrome
Source: BMC Genomics. 2022 Jun 21;23:458. doi: 10.1186/s12864-022-08693-4 (PMC9210747; doi:10.1186/s12864-022-08693-4)
Supplement: Supplementary file 4 — Additional file 4: Supplementary File 2. Input file for DMLE software for age of variant analysis. [file 12864_2022_8693_MOESM4_ESM.pdf]

Supplementary File 2: Input file for DMLE software for age of variant analysis.

```
Data as genotypes? Yes = 1, No = 0
0
Genetic model: Dominant=0,Recessive=1
1
Read old file?: (0=no, 1=yes):
0
Use fixed random seed?:(0 = no (=random), negative integer = yes
(=fixed), and is the seed):
0
# chromosomes (N):
13
# loci per chromosome (L):
4
Numbers of haplotypes in the normal(base) pop.:
27      1       1       1       1
2       3       3       1       1
2       3       2       1       1
13      2       2       1       1
4       2       2       2       1
1       3       2       2       2
2       3       3       2       1
2       1       1       2       1
2       2       1       1       1
2       2       2       2       2
1       1       1       2       2
2       2       1       2       1
1       2       3       1       1
1       1       1       1       2
Map distances:
0.0     0.000154 0.027627 0.028317
Run simulation?:
0
Mutation location (only used for simulated data):
0.028317
Mutation's low and high boundaries
-0.05 0.05
# simultaneous runs:
2
Starting value(s) for recdist. for each simul. run (-99 for random):
-99
Population growth rate:
0.01
Proportion of population sampled:
0.00248
Iterate ancestral states,mutation age,mutation location, allele
freq. (0=no, 1=yes):
1 1 0 1
Flip (potentially) all loci? (0=no, 1=yes):
1
Adjustment level for tree, recdist, ancestral, and internal
states,alleles:
```

```
1.0     3.0 0.005       0.5     0.5 0.1 1
Burn-in iterations:
1000000
Iterations:
1000000
Screen update and file update intervals:
100 100 1 0
Number of histogram bars:
200
Alpha level for recdist histogram:
0.05
Mutation age (-99 for random):
100
Mutation age boundaries:
0 1000
Star genealogy (0=no, 1=yes):
0
Loci for the root  (1xL) (-99):
-99
Frequency, and loci for the tip chromosomes (?x(L+1)):
10      3       3       3       3
3       2       2       2       2
Use sequence weights?
0
Weights for exons,introns,non-genes
1 0.17 0.02
Input file:
ncbi.txt
301500
301700
303234
311111
312456
```
